# Exploring the links of skeletal muscle mitochondrial oxidative capacity, physical functionality, and mental well-being of cancer survivors

**DOI:** 10.1038/s41598-024-52570-x

**Published:** 2024-02-01

**Authors:** Stephen G. Gonsalves, Leorey N. Saligan, Christopher M. Bergeron, Philip R. Lee, Kenneth W. Fishbein, Richard G. Spencer, Marta Zampino, Xinyi Sun, Jennifer Yeong-Shin Sheng, Vered Stearns, Michael Carducci, Luigi Ferrucci, Nada Lukkahatai

**Affiliations:** 1 National Institute of Nursing Research, National Institutes of Health, Bethesda, MD 20892 USA; 2 Laboratory of Clinical Investigation, Intramural Research Program, National Institute On Aging, National Institutes of Health, Baltimore, MD USA; 3 Department of Internal Medicine, University of Maryland, Baltimore, MD USA; 4 School of Nursing, Johns Hopkins University, Baltimore, MD USA; 5 School of Medicine, Johns Hopkins University, Baltimore, MD USA

**Keywords:** Physiology, Psychology, Medical research, Oncology

## Abstract

Physical impairments following cancer treatment have been linked with the toxic effects of these treatments on muscle mass and strength, through their deleterious effects on skeletal muscle mitochondrial oxidative capacity. Accordingly, we designed the present study to explore relationships of skeletal muscle mitochondrial oxidative capacity with physical performance and perceived cancer-related psychosocial experiences of cancer survivors. We assessed skeletal muscle mitochondrial oxidative capacity using in vivo phosphorus-31 magnetic resonance spectroscopy (^31^P MRS), measuring the postexercise phosphocreatine resynthesis time constant, τPCr, in 11 post-chemotherapy participants aged 34–70 years. During the MRS procedure, participants performed rapid ballistic knee extension exercise to deplete phosphocreatine (PCr); hence, measuring the primary study outcome, which was the recovery rate of PCr (τPCr). Patient-reported outcomes of psychosocial symptoms and well-being were assessed using the Patient-Reported Outcomes Measurement Information System and the 36-Item Short Form health survey (SF-36). Rapid bioenergetic recovery, reflected through a smaller value of τPCr was associated with worse depression (*rho* ρ = − 0.69, *p* = 0.018, and Cohen’s d = − 1.104), anxiety (ρ = − 0.61, *p* = .046, d = − 0.677), and overall mental health (ρ = 0.74, *p* = 0.010, d = 2.198) scores, but better resilience (ρ = 0.65, *p* = 0.029), and coping-self efficacy (ρ = 0.63, *p* = 0.04) scores. This is the first study to link skeletal muscle mitochondrial oxidative capacity with subjective reports of cancer-related behavioral toxicities. Further investigations are warranted to confirm these findings probing into the role of disease status and personal attributes in these preliminary results.

## Introduction

Cancer treatment can have both short- and long-term impact on quality of life. Functional impairment, characterized by fatigue, physical inactivity, and reduction in daily productivity^1,2^ and often assessed using validated patient-reported outcome (PRO) measures such as the Patient-Reported Outcomes Measurement Information System (PROMIS) questionnaires^3^, is one of the most common outcomes of cancer studies. No consistent therapeutic strategy has been established for cancer treatment-related functional impairment, in part because the etiology of this condition remains poorly understood^4^. Previous reports have correlated cancer treatment-related physical functional impairments with decreases in muscle mass and strength^5,6^. These declines in skeletal muscle function have been attributed to impairment of skeletal muscle oxidative function in cancer patients, similar to what has been observed in the older population^7,8^.

Skeletal muscle mitochondrial energetics has been used to explain impairment in physical mobility and walking performance in aging adults. Phosphorus magnetic resonance spectroscopy (^31^P-MRS) has been used effectively for the non-invasive investigation of skeletal muscle metabolism under various physiological conditions^9–12^. Following exercise cessation, the phosphocreatine recovery rate, τPCr, as measured by ^31^P-MRS, serves as a marker of oxidative metabolism, with a smaller value of τPCr reflecting more rapid metabolic recovery. For example, τPCr has been correlated with age-related performance of walking tasks^13–15^. In addition, there is also evidence to suggest that a rapid bioenergetic recovery rate, as measured by τPCr, may be associated with muscle strength^14^.

The τPCr, referred to as the PCr recovery time constant, has been used in studies as a measure of muscle metabolism and oxidative capacity due to its non-invasive assessment, sensitivity to muscle metabolism, reflection of mitochondrial function, clinical relevance, and predictive value in assessing muscle recovery in different populations^16–18^. The term "tau (τ)" refers to the time constant of the recovery curve, which reflects the rate of PCr regeneration. A faster τ indicates a greater rate of PCr recovery and thus better muscle oxidative capacity.

While there are validated objective measures available, the assessment of cancer-related physical impairment continues to face challenges due to the lack of guidelines on the standardized assessment of physical function in cancer patients. Hence, the multifactorial etiology of physical impairment in cancer, stemming from the disease and its treatments, contributes to ongoing research efforts to better understand and address these complexities^19^. Hence, the aims of this exploratory project were to evaluate skeletal muscle oxidative capacity in cancer survivors who received chemo or hormone therapy, and secondarily to explore the relationships between skeletal muscle oxidative capacity with physical performance (i.e., balance, coordination, and lower limb muscle strength) and perceived cancer-related physical (i.e., pain, fatigue) and psychosocial experiences (i.e., mental health, depression, anxiety, resilience, and coping). We hypothesized that measures of skeletal muscle oxidative capacity correlate with subjective and objective reports of physical impairments among cancer survivors.

## Methods

### Sample recruitment and ethical considerations of the study

To answer the study goals, an ongoing parent study titled, “Effectiveness of the combined technology-enhanced home exercise program and other non-pharmacological interventions on fatigue, physical function, and well-being among cancer survivors” (NCT 03576274) was amended and was approved by the Institutional Review Board of the Johns Hopkins University Hospital. All methods were performed in accordance with the institution’s relevant guidelines and regulations in the author affiliations. Study participants were referred from oncology outpatient clinics at Johns Hopkins University Hospital for these parent and exploratory studies. During phone screening, participants without a diagnosis of substance use disorder in the last five years and contraindications to MRS (e.g., metal implants, devices, prosthesis, claustrophobia), and not pregnant or lactating, were invited to participate in the exploratory study. Eleven cancer survivors from the parent study were enrolled in this exploratory ^31^P-MRS study from September 2020 to February 2022. Of these 11 cancer survivors, 45% completed cancer treatment for at least three months prior to data collection and 55% received immunotherapy during participation. Informed consents were obtained prior to performing any study measures in both parent and exploratory studies.

### Measures

Participants underwent a series of physical assessments using the short physical performance battery (SPPB). The SPPB consisted of three timed motor tests (tandem tests, five timed sit-to-stand chair stands, and a usual-pace gait speed measurement) to assess several motor domains including static and dynamic balance, coordination, and indirectly, lower-limb muscle strength^20^. The scores from each SPPB test were combined to give an overall score ranging from 0 to 12, with higher scores indicating better physical performance. The SPPB were conducted in one setting, in a private room, prior to scheduling the ^31^P-MRS muscle scan.

To assess perceived cancer-related physical and psychosocial experiences, a battery of validated questionnaires were administered prior to ^31^P-MRS muscle scan: (1) PROMIS® -29^3^ with health domains for depression, physical function, anxiety, fatigue, sleep, social role and pain; (2) the 36-Item Short Form health survey (SF-36)^21^ with health domains for physical emotion, body pain, mental health, social function, and general health; (3) the Connor–Davidson Resilience Scale (CD-RISC)^22^, and (4) the Coping Self-Efficacy Scale 13 (CSE-13)^23^. High scores indicate worse psychopathology for the negatively worded concepts for PROMIS®-29 (pain, depression, fatigue, and anxiety), but better functioning for the scales and subscales of the SF-36, CD-RISC, and the CSE-13.

### Skeletal muscle oxidative capacity

The ^31^P-MRS skeletal muscle oxidative capacity measurement protocol was previously described by Choi et al.,^13^ where a 3 T Philips Achieva MR scanner (Philips, Best, The Netherlands) and a 10-cm ^31^P-tuned transmit-receive surface coil (PulseTeq, Surrey, UK) fastened over the left vastus lateralis muscle estimated skeletal muscle phosphorus-containing metabolites. Participants performed a rapid ballistic knee extension exercise while lying supine in the machine^13,24^. Multiple measurements were taken before, during, and after the exercise, using radio frequency excitation pulses. The data collected had a temporal resolution of 6 s over a total scan duration of 7.5 min. The exercise duration was controlled to reduce the PCr peak height by 33–66%, not exceeding 42 s. The recovery period after the exercise varied from 5.8 to 6.3 min depending on the exercise duration. The collected spectra were processed and quantified using JMRUI software (version 5.0) and a nonlinear least squares algorithm^25–27^.

#### *Skeletal muscle oxidative ATP resynthesis rate determined by *^*31*^*P-MRS*.

Calculating the recovery rate for phosphocreatine was done by fitting the postexercise time-dependent change in PCr to a mono-exponential function of the form:

$${\text{PCr}}\left( {\text{t}} \right) \, = {\text{ PCr}}_{0} + \, \Delta {\text{PCR}}\left( {{1 }{-}{\text{ exp }}\left( { - {\text{t}}/{\text{ t}}_{{{\text{PCr}}}} } \right)} \right)$$

where PCr_0_ is the end-of-exercise, PCr peak area, ΔPCr is the decrease in peak area from its pre-exercise baseline value of PCr_baseline_ to PCr_0_, and t_PCr_ is the exponential recovery time constant for PCr.

This oxidative capacity measurement serves as an indicator of the skeletal muscle's capacity for oxidative phosphorylation, the process of producing energy using oxygen. At resting period post exercise, when energy demands are minimal, the resynthesis of phosphocreatine primarily relies on the maximum production of ATP by the mitochondria, with little or no contribution from anaerobic metabolism^28–32^. To find the percentage of phosphocreatine depletion, we calculated the decrease in the peak area of phosphocreatine from its baseline level before exercise (PCr baseline) to the highest-level right after exercise (PCr_0_). We also monitored the pH level inside the skeletal muscle to prevent acidosis, calculated based on the chemical shift of inorganic phosphate (Pi) in relation to phosphocreatine. The goal was to ensure that the intramuscular pH did not drop below 6.80, to avoid acidosis^28^.

### Statistical analyses

Descriptive statistics described the demographic and clinical characteristics of the study participants and t-tests compared mean scores for all continuous variables. Spearman *rho* correlations explored the relationships of the main study outcome variable (τPCr) with the variables of physical performance and patient reported outcome (PRO) survey results (Table 2). The PRO responses from the study participants were also compared with available normative values for reference. Many studies have used the U.S. general population normative reference to compare the effectiveness of different treatments or assess the overall prognosis of cancer patients^33–35^, as well as the cancer survivor’s psychological response to disease outcomes^36^.

All analyses were performed with SPSS for Mac (IBM SPSS® Software, version 29.0). Statistical significance for correlations and t-tests was defined at the *p* < 0.05 level, two-tailed. The exploratory nature of this project hoped to observe trends; hence, multiple corrections were not conducted. Nevertheless, boot strapping (BCa) methods for all significant Spearman *rho* (*p* < 0.05) correlations were performed (total samples 5,000; confidence interval level = 95%) to confirm significance^37^. The dataset used and analyzed during the current study is available from the corresponding author upon reasonable request.

## Results

### Sample description

#### Patient demographics

As shown in Table 1, the age of the 11 study participants ranged from 34 to 70 years (M ± SD; 53.27 ± 12.73). The participants were nearly equally divided between males (45.5%) and females (54.5%). Most participants identified as White (72.7%). The study participants were diagnosed with seven cancer types, with four participants (36%) diagnosed with breast cancer. Of the 11 study participants enrolled, all had received some combination of treatment for their cancer: about a third had received hormone therapy (27.3%), nearly half received chemotherapy, immunotherapy (45.5/54.5%), and radiation (45.5%), and over half had surgery (63.6%). The average time since cancer diagnosis at time of study enrollment was 5.91 years (± 4.59). Cancer type, status, and treatment are also reported in Table 1.

**Table 1:** Demographics of Study Population.

Characteristic	N = 11
Age	53.27 $\pm$ 12.73
Sex	
Female	6 (54.5%)
Male	5 (45.5%)
Marital status	
Married	7 (63.6%)
Never married	2 (18.2%)
Other*	2 (18.2%)
Race	
White	8 (72.7%)
Other	3 (27.3%)
Ethnicity	
Non-Hispanic/Latino	10 (90.9%)
Not reported	1 (9.1%)
Cancer type	
Breast cancer	4 (36.4%)
Prostate cancer	2 (18.2%)
Other^†^	5 (54.5%)
Years since cancer diagnosis	5.91 $\pm$ 4.59
Cancer treatment type	
Chemotherapy	6 (45.5%)
Radiotherapy	6 (45.5%)
Surgery	7 (63.6%)
Hormonal therapy	3 (27.3%)
Immunotherapy	5 (54.5%)

Plus-minus values are means $\pm$ standard deviation. * Other = includes

domestic partnership and widowed. † Other = cancer types that include melanoma, lymphoma, mesothelioma, cancer of chest/liver/spleen, and renal cell carcinoma.

### Reports of physical function and psychosocial experiences

Tables S1 and S2 (see supplemental material) also describe the physical performance test results. There were no significant correlations between SPPB results and τPCr (*p* = 0.357). In addition, the SPPB battery results did not show any significant difference between the sexes (*p* = 0.832). However, there was a significant difference (*p* = 0.041) in the SPBB between years since cancer diagnosis (≤ 5-years, ≥ 6-years). Both male and female participants in the current study exhibited significantly (*p* < 0.000) lower mean SPPB scores (Female: 10.20, Male: 10.67) compared to the reference values (Female: 11.8, Male: 11.9)^38^.

For PROs, SF-36 assessments (higher scores signify better self-reported health) of study participants were slightly better than the U.S. general population (50 ± 10)^39^, including physical function (80.0 ± 22.02) and general mental health (73.45 ± 18.00). For the PROMIS®-29 assessments, the study participants had slightly worse symptom scores than the general U.S. population (norm of 50)^40^, particularly, worse anxiety (54.24 ± 10.13), sleep (54.41 ± 8.96), and fatigue (52.59 ± 11.47). However, the study participants’ PROMIS®-29 fatigue scores were lower (less fatigued) compared to the PROMIS®-29 fatigue scores of a large, population-based sample of patients with recently diagnosed cancer–and signs of advanced disease (52.59 versus 55.8, respectively)^41^. The study participant’s level of resilience (31.18 ± 4.38)^36^, were similar with the general U.S. population (31.78 ± 5.41), but higher than the resilience of 169 Spanish women who had undergone surgery for breast cancer (25.35 ± 7.36)^42^. The level of coping for our study population (96.91 ± 18.57) was lower than the original survey’s validation study population of 348 participants HIV-seropositive men (137.4 ± 45:6)^23^.

### Skeletal muscle mitochondrial oxidative capacity

The mean τPCr (rapid bioenergetic recovery) for the 11 study participants (Table 2) was M = 50.53 s (SD = 13.55 s). The difference in mean τPCr between the sexes was not significant (*p* = 0.291).

**Table 2:** Correlations of τPCr and Study Variables. Correlations between τPCr levels measured by ^31^P Spectroscopy and physical performance metrics, as well as patient-reported outcomes, providing an analysis of their associations in the study population.

Test variables (N = 11)	*M*	*SD*	ρ	BCa†		*p* value
				Lower	Upper	
τPCr*	50.53	13.55	–	–	–	–
Age	53.27	12.73	0.54	− 0.251	0.972	0.089
Short Physical Performance^£^	10.55	1.92	− 0.31	− 0.825	0.394	0.357
PROMIS-29^±^						
Depression	49.16	6.95	− *0.69*	− *0.896*	− *0.266*	*0.018*
Physical function	49.48	9.18	− 0.27	− 0.816	0.471	0.426
Anxiety	54.24	10.13	− *0.61*	− *0.973*	*0.273*	*0.046*
Fatigue	52.59	11.47	− 0.33	− 0.942	0.453	0.322
Sleep	54.41	8.96	− 0.17	− 0.733	0.435	0.619
Social Role	52.28	9.14	0.83	− 0.576	0.826	0.809
Pain	51.30	8.49	− 0.19	− 0.917	0.571	0.563
SF-36^∓^						
Physical Function	80.00	22.02	− 0.23	− 0.712	0.388	0.504
Physical Role	81.25	22.16	0.59	− 0.350	0.981	0.122
Emotion	76.63	31.63	0.32	− 0.296	0.780	0.364
Body Pain	65.45	25.20	− 0.14	− 0.632	0.661	0.968
Vitality	47.08	20.98	0.31	− 0.608	0.948	0.383
Mental health	73.45	18.00	*0.74*	*0.429*	*0.905*	*0.010*
Social Function	54.55	28.65	0.034	− 0.763	0.748	0.914
General Health	49.64	22.18	0.17	− 0.615	0.887	0.610
**Connor–Davidson resilience**^†^						
Total Resilience	31.18	4.38	*0.65*	*0.071*	*0.935*	*0.029*
Coping Self-Efficacy-13^š^						
Coping Self-Efficacy	96.91	18.57	*0.63*	*0.056*	*0.960*	*0.039*

Study variables, significance, and abbreviations: *M* = mean, *SD* = standard deviation, ρ = Spearman correlation coefficient, p-value = *p* < .05., 2-tailed. Italicized and numbered (1–4) p-values = bootstrapped p-values with bias corrected and accelerated (BCa) confidence intervals. Bootstrap specifications sampling method = simple, with total number of samples used in the bootstrapping procedure = 5,000; confidence interval level = 95%; BCa significant p-values (*p* < .05).

* = τPCr recovery is a measure of muscle metabolism used to assess skeletal muscle oxidative capacity. The measure involves the use of magnetic resonance spectroscopy to monitor the recovery of phosphocreatine (PCr) levels in muscle after exercise. The term "tau" refers to the time constant of the recovery curve, which reflects the rate of PCr regeneration. A smaller value of tau indicates a greater rate of PCr recovery and better muscle oxidative capacity.

£ = Short Physical Performance Battery is (SPPB) assesses physical function and mobility in older adults. The SPPB consisted of three timed motor tests to assess static/dynamic balance, coordination, and lower-limb muscle strength. The scores from each test are combined to give an overall score ranging from 0 to 12, with higher scores indicating better physical performance.

±  = Patient-Reported Outcomes Measurement Information System (PROMIS), version 29, U.S.A. mean = 50 (SD 10). Increase scores are better for positive worded concepts and worse for negatively worded concepts.

∓  = 36-Item Short Form (SF-36) Health Survey score metric for U.S.A. has a mean = 50 (SD 10) with increased scores indicating better outcomes.

^†^ = 10-Item Connor–Davidson Resilience (CD-RISC-10) for U.S.A. with increased score indicating a better outcome.

Š = Coping Self-Efficacy Scale (CSE-13**)** scale consists of 13 items measuring perceived ability to cope with stress. Higher scores indicating greater perceived coping self-efficacy.

### Correlates of skeletal muscle mitochondrial oxidative capacity

A shorter τPCr recovery time, indicating better skeletal muscle oxidative capacity, was correlated with high (worse) PROMIS® depression (ρ = − 0.69, *p* = 0.018) and anxiety scores (ρ = − 0.63, *p* = 0.046), but low (worse) mental health score (ρ = 0.74, *p* = 0.010) (Table 2). However, faster τPCr recovery time was also correlated with high (better) resilience (ρ = 0.65, *p* = 0.0294) and coping-self efficacy scores (ρ = 0.63, *p* = 0.04). In summary, a faster τPCr recovery rate in skeletal muscle is associated with high severity in depression and anxiety, worse mental health state, but higher resilience and coping. Boot strapping reinforced significant findings for PROMIS®-29 depression at BCa lower -0.896, upper -0.266, SF-36 mental health at BCa lower 0.429, upper 0.953; and Connor–Davidson total resilience at BCa lower 0.071, upper 0.935.

## Discussion

This is the first study to explore the links of skeletal muscle oxidative capacity with reports of psychophysical impairments from cancer survivors. Of all the demographic and clinical characteristics of the study sample, years since cancer diagnosis was the only demographic variable that significantly categorized study participants based on objective physical performance tasks and subjective physical impairment reports. Study participants who received cancer diagnoses > 6 years performed better with the SPPB tasks and reported better SF-36 physical function than those who were diagnosed < 5 years (*p* = 0.041 and 0.013, respectively). This may be related to the acute neurotoxic effects of cancer therapy. However, we observed lower SPPB test scores (10.55 ± 1.92) and higher PROMIS 29 physical function subscale scores (49.48 ± 9.18) suggesting the usual discrepancy between objective measurements and subjective reporting of physical abilities, as similarly observed in previous studies^43,44^.

Notably, individuals with less than or equal to five-years since cancer diagnosis reported slightly better physical function compared to the overall study population, but this did not reach statistical significance. In contrast, those with over six-years since cancer diagnosis showed a significantly higher self-perceived physical function than the overall population (see supplemental material, S2), suggesting a potential positive adaptation to long-term survivorship, where individuals may develop a more optimistic view of their physical capabilities over time. Possible contributing factors may include the gradual amelioration of treatment-related side effects and the cultivation of effective coping mechanisms and resilience. The consistent global mental health scores across both subgroups imply that, regardless of survivorship duration, mental well-being is still a resilient aspect of cancer survivorship. These observations are consistent with prior reports that underscore the persistent psychological resilience of individuals post-cancer treatment^45,46^. Our findings underscore the dynamic nature of survivorship experiences and advocate for personalized interventions that consider the evolving needs of individuals at different stages of their cancer journey.

The main distinctive finding of this exploratory study was that rapid τPCr recovery rate was consistently associated with poor mental health state (e.g., PROMIS -29 depression, anxiety, and the SF-36 mental health scores), which is opposite to what is currently reported from a very limited literature suggesting positive links between better muscle oxidative capacity and perceived lower levels of depression and anxiety^47–50^. These discrepancies may be related to the procedures used in assessing muscle oxidative capacity. In our study, we used τPCr, a validated measure of estimating maximum skeletal muscle mitochondrial ATP production with no or minimal contribution of anaerobic metabolism^23–30^, using a widely tested ^31^P-MRS muscle oxidative capacity measurement protocol^9^. Previous studies have used methods such as ex vivo biochemical assays to measure oxidative phosphorylation, ATP production, and oxygen consumption to estimate mitochondrial function^48^. In contrast, ^31^P-MRS measurements–as used in this study and in the Brown et al., 2019 study–provide a metric of in vivo mitochondrial metabolism^49,51^. However, the 2019 study by Brown and colleagues had results that differed from this study. Their study found that declining skeletal muscle oxidative capacity in older adults is associated with clinically significant depressive symptoms at follow-up.

Our findings may also suggest that physiology and metabolism may be driving the mental state of our study participants. There are several factors that can explain these observations. Robust skeletal muscle oxidative capacity has been linked with higher levels of physical function and physical activity, as well as reduced fatigability^52–56^. However, these improvements in physical performance do not always lead to, or consistently correspond with, concurrent improvements in well-being, including symptoms of depression, anxiety, or cognitive fatigue^57–60^. This discrepancy may be related to several meachanism such as behavioral hyperactivity which we hypothesize as a coping mechanism to overcome energetic deficiency from hypoxia or starvation.

Advancements in cancer therapeutics have strategically targeted cellular metabolism, aiming to deprive and starve cancer cells of oxygen and essential nutrients^61^. A noteworthy example involves the use of creatine kinase targeting as an anti-cancer strategy, effectively inhibiting tumor growth and directly influencing creatine metabolism and mitochondrial function^62^. Importantly, our study sheds light on the potential consequences of these therapeutic approaches, especially in inducing hypoxic cellular states. MRS has demonstrated that hypoxic conditions trigger an appreciable drop in cellular maximum oxygen consumption, leading to the release of excitatory metabolites such as glutamate and glutamine, along with a reduction in inhibitory metabolites like GABA^63^, which could be a similar consequence with hypoxic states triggered by cancer and its treatments. In contrast, correction of ischemia showed the opposite effect by increasing the level of inhibitory metabolites detected by MRS^64^, and eliciting depressive behaviors such as social apathy and poor appetite^63^. This observed metabolic shift may be particularly relevant in understanding the potential impacts of hypoxic states induced by cancer and its treatments on the psychophysical well-being of cancer patients.

High energy phosphate metabolism, PCr-ATP buffering and the creatine kinase system play important roles in maintaining constant ATP levels for proper human brain functioning^65^. Although the precise mechanisms remain unclear, creatine and PCr have been reported to have neuroprotective effects in certain brain disorders including ischemic stroke and Alzheimer’s disease^65^. Therefore, our finding of the associations of rapid bioenergetic recovery rate, τPCr, with changes in the PRO scores that reflected a reduction in the mental health state, may be linked to the increased susceptibility of our cancer survivors to behavioral toxicities related to the effects of their cancers and cancer treatments to cellular metabolism.

The relationship between resilience and depression has been widely studied. One proposed assumption asserts that the resilient diathesis against depression is relatively stable but dynamic, and could be enhanced^66^. A prior longitudinal study on children showed that some children were overwhelmed by strong stresses at some point in their life causing a consequential reduction in resilience, but they were able to recover in later life and be resilient as much as those who were resilient the whole way through^67^. In our study population, the opposite relationships of depression/anxiety and resilience/coping with rapid skeletal muscle recovery may be related to the stresses of dealing with cancer and its treatments. For breast cancer survivors, experiences of depression and anxiety are common and can be related to the stage of cancer, the type of cancer treatment they received or receiving, and dealing with the adverse effects of these treatments, the changes in femininity, sexuality, and role performance, or fear of recurrence, among others^68–70^. However, resilience has been reported to be high among breast cancer survivors even when experiencing adversities, confirming the hypothesis that breast cancer survivors perceive themselves as effective in attending to, understanding, and repairing their emotions^71,72^. In summary, patients can be depressed related to their illness, but that does not mean that they are not resilient. More importantly, the dynamic relationships between depression and resilience in cancer patients may be different than the larger literature involving non-medically ill patients and will require future study.

## Limitations

The study was limited by the exploratory nature of the project, namely having a small sample size, enrolling heterogeneous cancer and treatment types, and a restricted range of race and ethnicity among study participants. The limited sample, inability to control for potentially confounding factors such as date since completion of cancer treatment, comorbidities, and concomitant medications may have led to type II statistical errors for the significant correlations; thereby, warranting cautious interpretation of the observed associations and highlighting the need for further research with a larger and more controlled dataset. Oxidative capacity recovery, as an outcome for this exploratory study, is limited by substrate and oxygen delivery^73^; so, future studies should exclude participants with medical conditions with potentially flow-limited vascular and neurological states such as peripheral vascular diseases (e.g., intermittent claudication, diabetes), cardiomyopathy (especially chemo-related cardiotoxicity^73^).

In addition, assessment of fatigability index could have further enriched the depth and clinical relevance of the results as a complementary measure to τPCr, offering additional information about the adaptive capacity of skeletal muscles in the context of cancer survivorship.

**Figure 1:**
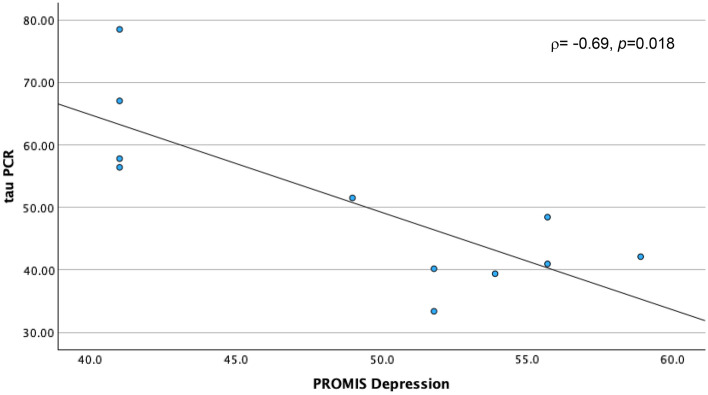
τPCr and Depression. Figure illustrates a scatter plot depicting the relationship between Tau PCr measurements obtained through Magnetic Resonance Spectroscopy (MRS) on the y-axis and Patient-Reported Outcome Measurement Information System (PROMIS-29) Depression Scores on the x-axis.

**Figure 2:**
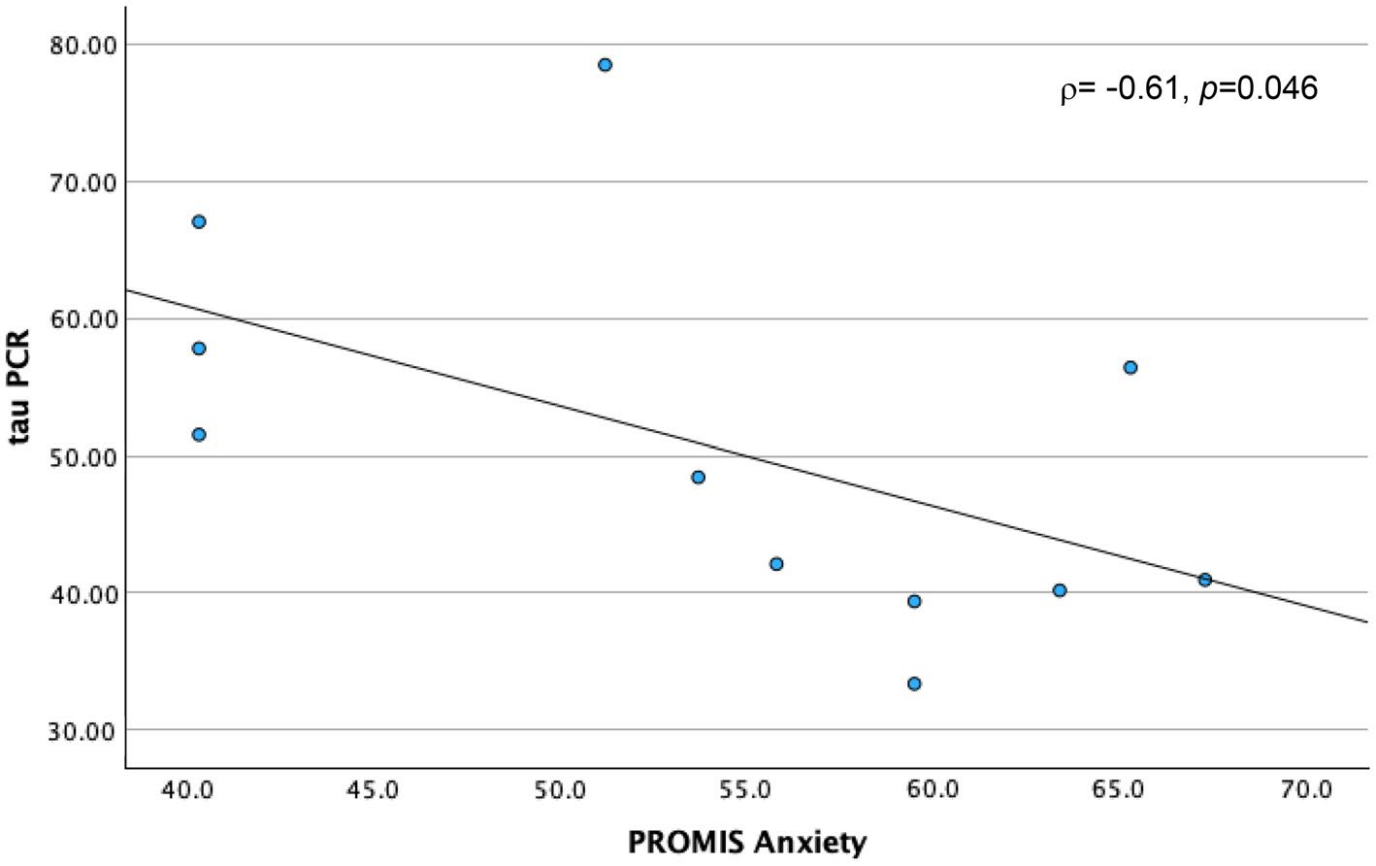
τPCr and Anxiety. Figure illustrates a scatter plot depicting the relationship between Tau PCr measurements obtained through Magnetic Resonance Spectroscopy (MRS) on the y-axis and Patient-Reported Outcome Measurement Information System (PROMIS-29) Anxiety Scores on the x-axis.

**Figure 3:**
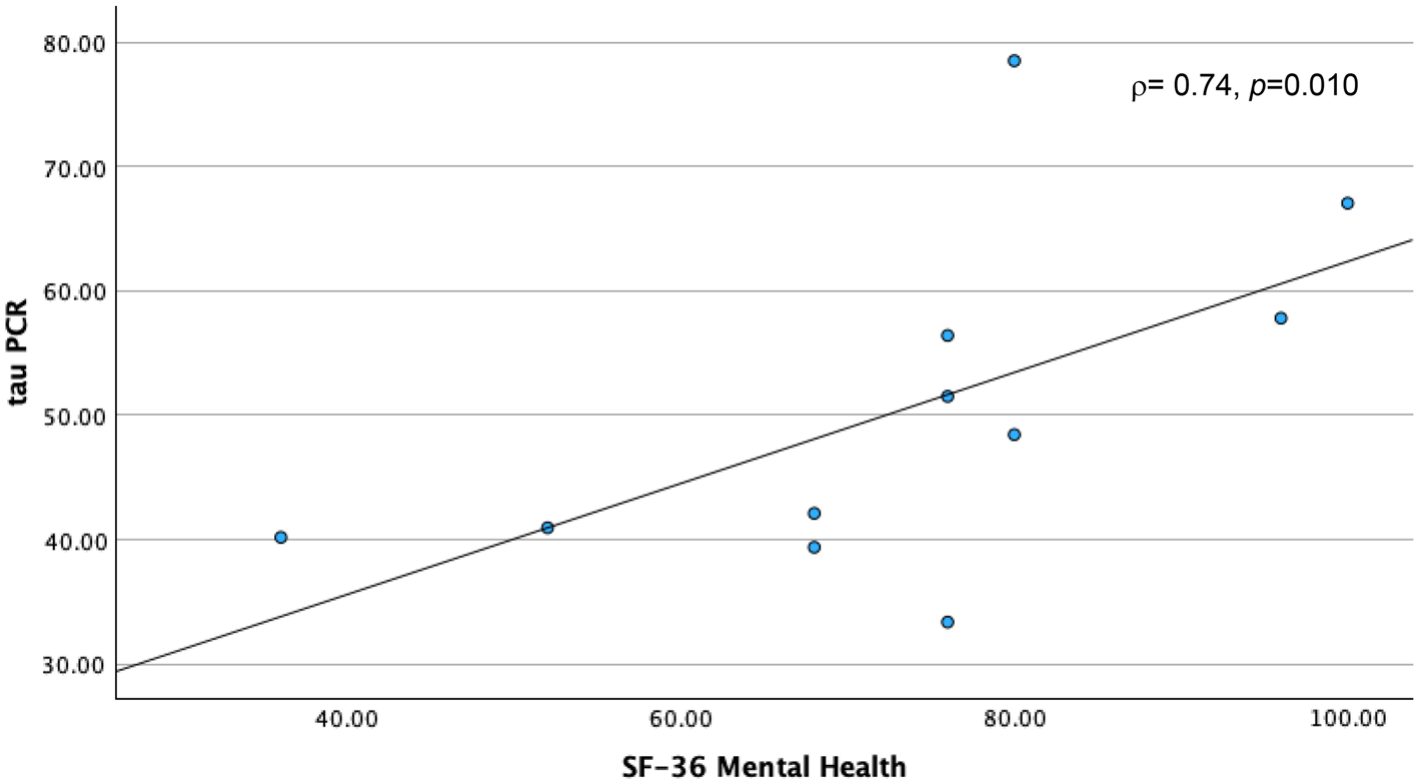
τPCr and Mental Health. A scatter plot depicting the relationship between Tau PCr Measurements obtained through Magnetic Resonance Spectroscopy (MRS) on the y-axis and Patient-Reported Outcome Survey results from 36-Item Short Form (SF-36) Mental Health Scores on the x-axis.

**Figure 4:**
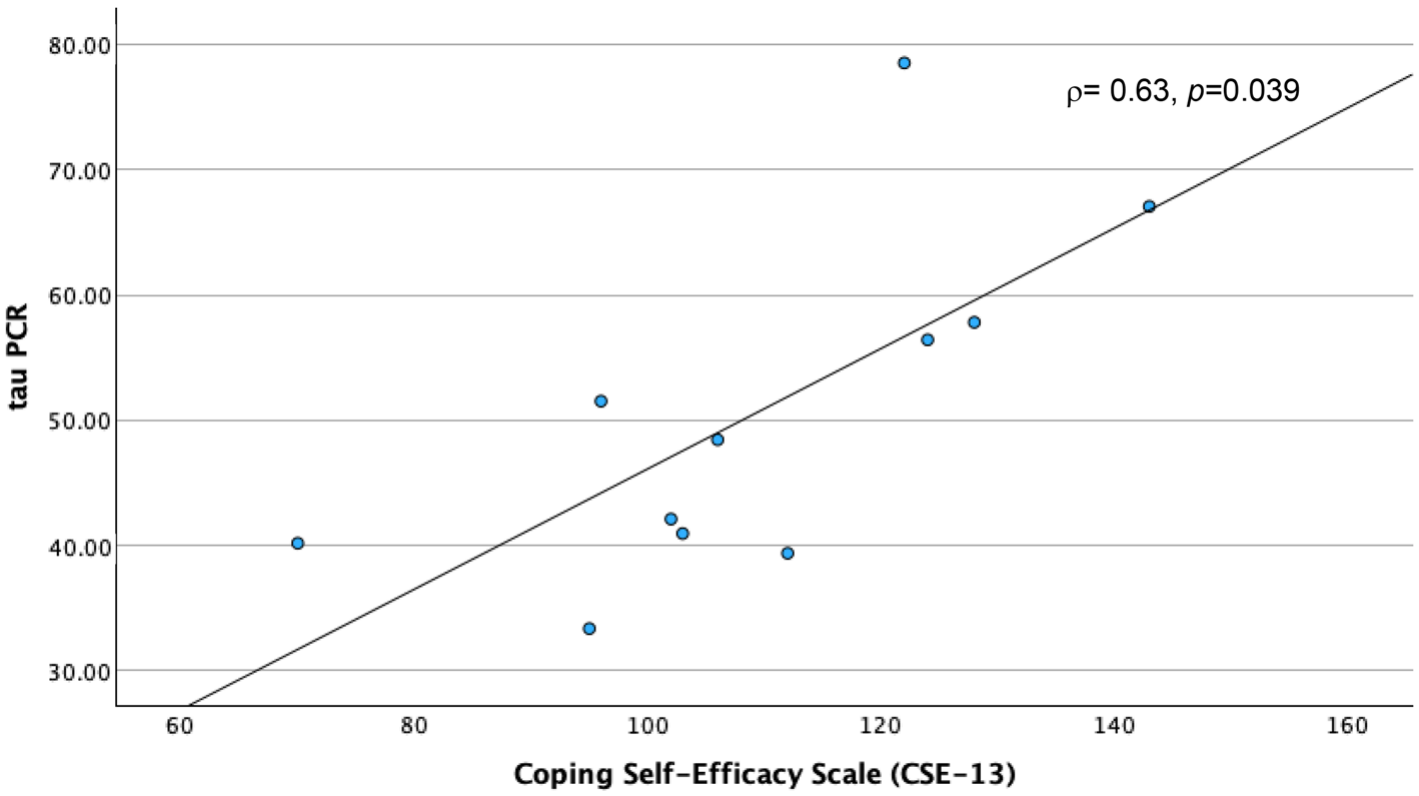
τPCr and Coping Self-Efficacy. A scatter plot depicting the relationship between Tau PCr Measurements obtained through Magnetic Resonance Spectroscopy (MRS) on the y-axis and Patient-Reported Outcome Survey results from the Coping Self-Efficacy (CSE-13) Scale on the x-axis.

**Figure 5:**
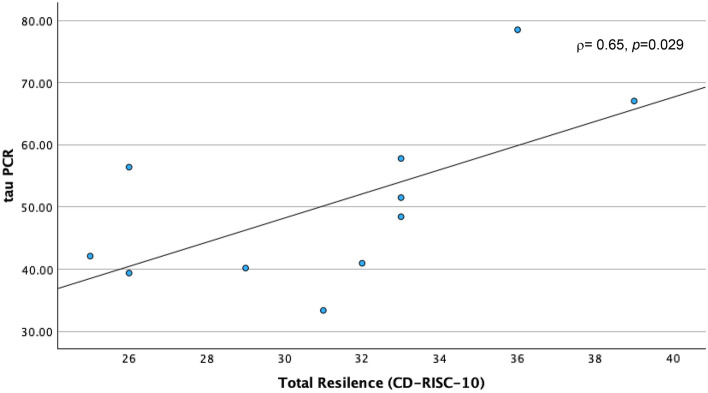
τPCr and Resilience. A scatter plot depicting the relationship between Tau PCr Measurements obtained through Magnetic Resonance Spectroscopy (MRS) on the y-axis and Patient-Reported Outcome Survey results from the Conner-Davidson Resilience Scale (CD-RISC-10) on the x-axis.

Furthermore, the selection of appropriate analyses is fairly limited to traditional tests (e.g., Shapiro–Wilk test for normality) and sample size dependent tests, so multiple corrections were not conducted. However, despite the relatively small sample size, significant (*p* ≤ 0.05) Spearman’s *rho correlations* (Figs. 1, 2, 3, 4, 5) remained significant by confirmatory boot strapping methods. Further, associations of τPCr were high with depression (ρ = − 0.69, *p* = 0.018, d = − 1.104), anxiety (ρ = − 0.61, *p* = 0.046, d = − 0.677), and mental health (ρ = 0.74, *p* = 0.010, d = 2.198).

Other limitations stemmed from the study design, including the influence of timing in measuring the PROs in cancer survivors or lack of controls to compare MRS τPCr analysis with patient reported outcome survey testing. It is important to note that physical impairment related to cancer and its treatment is a complex and multi-faceted issue, and further research is needed to fully understand its etiology, and hopefully, identify potential therapeutic targets.

## Conclusions

This present study shows that inefficiency in mitochondrial oxidative capacity is linked to subjective measures of mental health among cancer survivors who received chemo, immune, or hormone therapies. These findings call for further investigations on the mechanisms of functional impairment in cancer patients, and the role of cellular metabolism on the physical and behavioral toxicities of cancer and its treatments. These findings may trigger promising new directions for studying both the mechanisms and possible clinical interventions to treat or manage functional impairments experienced by cancer patients.

### Supplementary Information

Supplementary Information.

## Data Availability

The datasets generated and/or analyzed during the current study are not publicly available because the human subjects study is still active but are available from the corresponding author on reasonable request.
