# Supplementary material for: Exploring the links of skeletal muscle mitochondrial oxidative capacity, physical functionality, and mental well-being of cancer survivors
Source: Sci Rep. 2024 Feb 1;14:2669. doi: 10.1038/s41598-024-52570-x (PMC10834492; doi:10.1038/s41598-024-52570-x)
Supplement: Supplementary file 1 — Supplementary Information. [file 41598_2024_52570_MOESM1_ESM.pdf]

Supplemental Material

Exploring the Links of Skeletal Muscle Mitochondrial Oxidative Capacity, Physical Functionality, and Mental Well-being of Cancer Survivors

Stephen G. Gonsalves[a]; Leorey N. Saligan[a],* Christopher M. Bergeron[c]; Philip R. Lee[c]; Kenneth W. Fishbein[c]; Richard G. Spencer[c]; Marta Zampino[e]; Xinyi Sun[b]; Jennifer Yeong-Shin Sheng[d] Vered Stearns[d]; Michael Carducci[d]; Luigi Ferrucci[c], Nada Lukkahatai[a,b]

Author Affiliations:
[a]National Institute of Nursing Research, National Institutes of Health, Bethesda, Maryland, USA
[b]School of Nursing, Johns Hopkins University, Baltimore, Maryland, USA
[c]Laboratory of Clinical Investigation, Intramural Research Program, National Institute on Aging, National Institutes of Health, Baltimore, MD, USA
[d]School of Medicine, Johns Hopkins University, Baltimore, Maryland, USA
[e]Department of Internal Medicine, University of Maryland, Baltimore, MD

Table 1S: Physical Performance Measures by Sex

Study's τPCr and Short Physical Performance Battery (SPPB)

Performance Measures (N=11)	Female		Male		
	M	*SD*	*M*	*SD*	*p* value
Tau PCr*	46.39	9.60	55.49	16.95	0.291
SPPB£	10.20	2.16	10.67	2.05	0.832

Study's SPPB by Sex, Comparison to Normative Values

Performance Measure	Current Study		Reference Values			Current Study		Reference Values		
	**Female** (6)		**Female** (1252)			**Male** (5)		**Male** (1044)		
	M	*SD*	*M*	*SD*	*p*	*M*	*SD*	*M*	*SD*	*p*
**SPPB£**	10.20	2.16	11.8	0.6	<0.000	10.67	2.05	11.9	0.6	<0.000

Study variables, significance, and abbreviations: *M* = mean, *SD* = standard deviation, p-value = *p*<.05, 2-tailed.

* = TPCr recovery is a measure of muscle metabolism used to assess skeletal muscle oxidative capacity. The measure involves the use of magnetic resonance spectroscopy to monitor the recovery of phosphocreatine (PCr) levels in muscle after exercise. The term "tau" refers to the time constant of the recovery curve, which reflects the rate of PCr regeneration. A faster tau indicates a greater rate of PCr recovery and better muscle oxidative capacity.

£ = Short Physical Performance Battery is (SPPB) assesses physical function and mobility in older adults. The SPPB consisted of three timed motor tests to assess static/dynamic balance, coordination, and lower-limb muscle strength. The scores from each test are combined to give an overall score ranging from 0 to 12, with higher scores indicating better physical performance.

Study mean age = 53.3 (±12.73); Female mean age = 48.5 (±13.19); Male mean age = 59.0 (±10.61).
SPPB normative values from Bergland and Strand, 2019 (BMC Geriatr. 2019, Aug 8;19(1):216. PMID: 31395008).

Female = mean total SPPB score for mean age 50.6 (±5.9) from Bergland et al. 95% confidence interval of this difference: From -2.1938 to -1.2062.
Male = mean total SPPB score for mean age 50.3 (±5.9) from Bergland et al. CI: 95% confidence interval of this difference: From -1.7691 to -0.6909.

The datasets generated and/or analyzed during the current study are not publicly available because the human subjects study is still active but are available from the corresponding author on reasonable request.

Table 2S: Mean And T-Test Comparisons of Study Variables and Patient Reported Outcome Scores by Years with Cancer Diagnosis.

**Years with Cancer Diagnosis (1-15 years), N=11**			
**Performance Measures**	**≤5 (n=6)<br>*M* (*SD*)**	**≥6 (n=5)<br>*M* (*SD*)**	***p* value**
Age	57.00 (11.08)	48.80 (14.34)	0.329
τPCr*	55.81 (14.88)	44.17 (9.48)	0.167
Short Physical Performance£	9.50 (2.07)	11.80 (0.45)	***0.041***
**PROMIS-29±**			
Depression	48.88 (8.71)	49.50 (5.06)	0.897
Physical Function	52.35 (15.60)	50.68 (6.33)	0.818
Anxiety	51.43 (10.24)	57.60 (9.99)	0.602
Fatigue	52.98 (15.16)	52.10 (6.43)	0.909
Sleep	50.25 (11.79)	55.00 (3.37)	0.383
Social Role	50.93 (11.41)	53.90 (6.31)	0.601
Pain	52.73 (9.68)	49.58 (7.52)	0.568
**SF 36 Scores∓**			
Physical Function	65.83 (20.59)	97.00 (4.47)	***0.013***
Role Physical	41.67 (49.16)	80.00 (20.92)	0.127
Emotion	66.66 (42.17)	73.33 (36.51)	0.788
Body Pain	60.17 (29.71)	71.80 (19.80)	0.238
Mental Health	74.33 (21.41)	66.40 (17.34)	0.523
Social Function	58.33 (34.16)	50.00 (24.39)	0.656
General Health	56.33 (27.08)	41.60 (12.88)	0.296
**Connor–Davidson Resilience Scale †**			
Total Resilience	33.00 (4.69)	29.00 (3.08)	0.138
**Coping Self-Efficacy-13š**			
Coping Self-Efficacy-13š	177.33 (16.51)	99.40 (20.37)	0.141

Study variables, significance, and abbreviations: *M* = mean, *SD* = standard deviation, and p-value = $p<.05$, 2-tailed.
* = τPCr recovery is a measure of muscle metabolism used to assess skeletal muscle oxidative capacity. The measure involves the use of magnetic resonance spectroscopy to monitor the recovery of phosphocreatine (PCr) levels in muscle after exercise. The term "tau" refers to the time constant of the recovery curve, which reflects the rate of PCr regeneration. A faster tau indicates a greater rate of PCr recovery and better muscle oxidative capacity.

£ = Short Physical Performance Battery is (SPPB) assesses physical function and mobility in older adults. The SPPB consisted of three timed motor tests to assess static/dynamic balance, coordination, and lower-limb muscle strength. The scores from each test are combined to give an overall score ranging from 0 to 12, with higher scores indicating better physical performance.
± = Patient-Reported Outcomes Measurement Information System (PROMIS), version 29, U.S.A. mean = 50 (SD 10). Increase scores are better for positive worded concepts and worse for negatively worded concepts.
∓ = 36-Item Short Form (SF-36) Health Survey score metric for U.S.A. has a mean = 50 (SD 10) with increased scores indicating better outcomes.
† = 10-Item Connor–Davidson Resilience (CD-RISC-10) for U.S.A. with increased score indicating a better outcome.
Š = Coping Self-Efficacy Scale (CSE-13) scale consists of 13 items measuring perceived ability to cope with stress. Higher scores indicate greater perceived coping self-efficacy.
